# Antcin K, a Triterpenoid Compound from *Antrodia camphorata*, Displays Antidiabetic and Antihyperlipidemic Effects via Glucose Transporter 4 and AMP-Activated Protein Kinase Phosphorylation in Muscles

**DOI:** 10.1155/2016/4867092

**Published:** 2016-05-08

**Authors:** Yueh-Hsiung Kuo, Cheng-Hsiu Lin, Chun-Ching Shih, Chang-Syun Yang

**Affiliations:** ^1^Department of Chinese Pharmaceutical Sciences and Chinese Medicine Resources, China Medical University, Taichung 40402, Taiwan; ^2^Department of Internal Medicine, Fengyuan Hospital, Ministry of Health and Welfare, Fengyuan District, Taichung 42055, Taiwan; ^3^Graduate Institute of Pharmaceutical Science and Technology, College of Health Science, Central Taiwan University of Science and Technology, No. 666 Buzih Road, Beitun District, Taichung 40601, Taiwan

## Abstract

The purpose of this study was to screen firstly the potential effects of antcin K (AnK), the main constituent of the fruiting body of* Antrodia camphorata*,* in vitro* and further evaluate the activities and mechanisms in high-fat-diet- (HFD-) induced mice. Following 8-week HFD-induction, mice were treated with AnK, fenofibrate (Feno), metformin (Metf), or vehicle for 4 weeks afterward. In C2C12 myotube cells, the membrane GLUT4 and phospho-Akt expressions were higher in insulin and AnK-treated groups than in the control group. It was observed that AnK-treated mice significantly lowered blood glucose, triglyceride, total cholesterol, and leptin levels in AnK-treated groups. Of interest, AnK at 40 mg/kg/day dosage displayed both antihyperglycemic effect comparable to Metf (300 mg/kg/day) and antihypertriglyceridemic effect comparable to Feno (250 mg/kg/day). The combination of significantly increased skeletal muscular membrane expression levels of glucose transporter 4 (GLUT4) but decreased hepatic glucose-6-phosphatase (G6 Pase) mRNA levels by AnK thus contributed to a decrease in blood glucose levels. Furthermore, AnK enhanced phosphorylation of AMP-activated protein kinase (phospho-AMPK) expressions in the muscle and liver. Moreover, AnK treatment exhibited inhibition of hepatic fatty acid synthase (FAS) but enhancement of fatty acid oxidation peroxisome proliferator-activated receptor *α* (PPAR*α*) expression coincident with reduced sterol response element binding protein-1c (SREBP-1c) mRNA levels in the liver may contribute to decreased plasma triglycerides, hepatic steatosis, and total cholesterol levels. The present findings indicate that AnK displays an advantageous therapeutic potential for the management of type 2 diabetes and hyperlipidemia.

## 1. Introduction

Diabetes mellitus hardly occurs in isolation but is most often part of an array of metabolic abnormalities that includes insulin resistance, hyperinsulinemia, and hypertriglyceridemia. The population of type 2 diabetes prevalence by 2025 will reach approximately 300 million [1]. Pathogenesis of type 2 diabetes has been proposed to display more than 90% of all diabetes mellitus patients [2]. Type 2 diabetes mellitus has revealed mechanisms of insulin resistance that target either impairs in *β*-cell function or insulin insensitive action at adipose tissue, skeletal muscle, or liver tissues.

**Figure 1:**
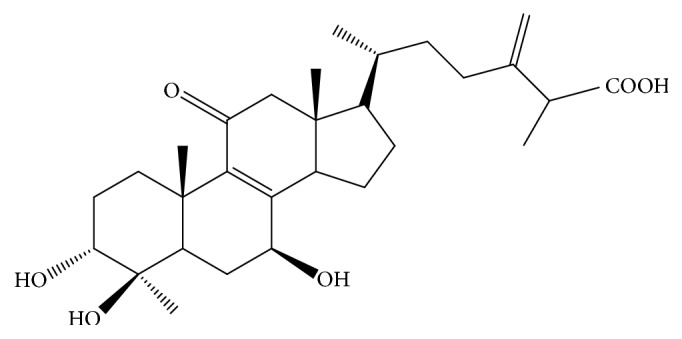
Chemical structure of antcin K (AnK).

*Antrodia camphorata* (Polyporaceae, Aphyllophorales) is edible as a folk remedy in the treatment of a variety of diseases in Taiwan. It is rare and expensive because it grows only on the inner heartwood wall of the endemic evergreen* Cinnamomum kanehirai*. The mycelia, filtrate of broth, and fruiting body of* A. camphorata* exhibit numerous physiological functions [3]. The fruiting body of* A. camphorata *consisted of terpenoids, such as antcins (A, B, and C), zhankuic acids (A, B, C, D, and E), 15*α*-acetyl-dehydrosulphurenic acid, dehydroeburicoic acid and dehydrosulphurenic acid, antcin E and F, methyl antcinate G and methyl antcinate H, and eburicoic acid. The solid culture of fruiting body and the filtrate in submerged culture have been shown to have hepatoprotective effects and antioxidant activities [4, 5]. Previous study had demonstrated that, in terms of* in vivo* metabolism, 13 terpenoids in* A. camphorata* were determined by using LC/MS/MS in rats plasma after oral administration, and plasma concentrations of ergostanoids were much higher than lanostanoids, and the ergostanoids underwent reduction and hydroxylation reactions* in vivo* [6]. Their mean residence time (MRT) ranged from 3 to 6 hr, and the lanostanoids were not active to metabolic reactions and were slowly eliminated with an MRT of 9–16 hr [6]. Antcin K (3*α*,4*β*,7*β*-trihydroxy-4*α*-methylergosta-8,24(28)-dien-11-on-26-oic acid, 2; AnK) (Figure 1), an active triterpenoid from the fruiting bodies of basswood cultivated* A. cinnamomea*, could induce apoptotic cell death in human liver cancer Hep3B cells [7]. Antcin K isolated from ethanol extracts of wild fruiting body has shown concentration-dependent (1–25 Mm) anti-inflammatory effects (by modulation of leukocyte activity and inhibition of ROS) induced by fMLP and TPA in human neutrophils [8, 9]. Our recent studies demonstrated that ergostatrien-3*β*-ol and dehydroeburicoic acid from* A. camphorata* exhibited an excellent antihyperglycemic and antihyperlipidemic activity [10, 11]. Nevertheless, the effects of antcin K, the main constituent of the fruiting body of* A. camphorata*, on diabetes and dyslipidemia are still unknown* in vitro* and in diet-induced diabetic rodents.

The glucose transporter 4 (GLUT4) has been regarded as a vital determinant of blood glucose homeostasis [12]. The elevated glucose levels, after huge caloric ingestions, are rapidly returned to normal. Insulin stimulates or contraction causes glucose uptake via eliciting translocation of GLUT4 from intracellular sites to the membrane [13, 14]. Levels of insulin-induced GLUT4 translocation in skeletal muscle of type 2 diabetic patients are markedly decreased [15]. Therefore, the improvement of GLUT4 levels or induced translocation may accelerate drug development. Peripheral glucose uptake into membrane of skeletal muscle could be promoted by two pathways including insulin-dependent mechanisms leading to Akt/PKB activation and contraction-regulated stimulation [16, 17] or hypoxia-regulated AMPK activation [17, 18]. AMPK play a dominant role in glucose and lipid metabolism. Since dysregulation of glucose and lipid catabolism in type 2 diabetes, AMPK activators would be promising therapies [19].

Metformin is used in the clinics as an antidiabetic drug in the management of type 2 diabetes [19] and it activates AMPK in both hepatocyte and skeletal muscle [19, 20].

Peroxisome proliferator-activated receptor *α* (PPAR*α*) plays a key role in regulation of lipid metabolism [21] and reduces circulating triglyceride (TG) concentrations via regulated numerous genes associated with lipogenic and fatty acids oxidation [22]. Fenofibrate is one of PPAR*α* agonists and has been used in the treatment of hypertriglyceridemia [23, 24].

The high-fat diet- (HFD-) fed C57BL/6J mouse could induce early type 2 diabetes and markedly increased adipose weights and produced resistance to insulin and increases in blood glucose, total cholesterol (TC), and TG levels [25–27]. Thus, this model was chosen to investigate both mechanistic activities and as a tool for developing novel therapeutic interventions [25]. Phosphorylation of Thr^172^ of *α* subunits is essential for AMPK activity [28]. This study was to screen firstly the potential effects of AnK* in vitro* and further to investigate the hypothesis that AnK could display the beneficial metabolic effects including antidiabetic and hypolipidemic effects by modulation of GLUT4 protein expression and activation of AMPK as compared with clinical drugs such as Metf and Feno; moreover, the targeted gene expressions were determined including PPAR*α* and fatty acid synthase (FAS) from the peripheral tissues of HFD-fed mice by the AnK treatment.

## 2. Materials and Methods

### 2.1. Chemicals

Antibodies of GLUT4 (number sc-79838) were obtained from Santa Cruz Biotech (Santa Cruz, CA, USA); phospho-AMPK (Thr^172^), PPAR*α* (number ab8934), and PPAR*γ* (number ab45036) were purchased from Abcam Inc. (Cambridge, MA, USA); FAS (number 3180), phospho-Akt (Ser473) (number 4060), total-AMPK (Thr^172^), and *β*-actin (number 4970) were from Cell Signaling Technology (Danvers, MA, USA). Secondary antibody anti-rabbit was from Jackson ImmunoRes. Lab., Inc. (West Grove, PA, USA).

### 2.2. Determination of the Active Compound

The fruiting body of* A. camphorata* was purchased from the Balay Biotechnology Corporation, Hsinchu City, Taiwan. A voucher specimen (CMPC393) was deposited at and identified by China Medical University. The fruiting bodies of AC (3.0 kg) were extracted three times with methanol and followed by chromatography using 50% ethyl acetate and 50% hexane. The procedure was as in a previously described report [29]. The purity of AnK is above 99%. Analytical instrument is the HPLC, SHIMADZU LC 20-A; the HPLC Column, TOSOH TSKgel DS-80Ts, and analytical condition, 100% MeOH.

### 2.3. Cell Culture

C2C12 skeletal myoblasts (ATCC, CRL-1772) were employed and performed as a previous report [11].

### 2.4. Detection of Expression Levels of Membrane GLUT4 and Phosphorylation of Akt (Ser473)* In Vitro*

The procedure was performed as a previous description [11, 30, 31]. Differentiated C2C12 cells were serum-starved in DMEM/BSA prior to incubation either with test compounds (AnK at 1, 5, 10, and 25 *μ*g/mL) or with vehicle for 30 min or with 100 nM insulin for 25 min, as previously described [32]. The homogenates were centrifuged and the pellet was resuspended and performed within membrane; protein concentration was analyzed via BCA assay (Pierce), and equal amounts of protein were then diluted four times in SDS sample buffer and subjected to SDS PAGE and were detected by Western blotting with antibodies specific for Akt, phospho-Akt Ser473, and GLUT4; and the analysis of density blotting was as in a previous report [11].

### 2.5. Animals and Treatments

The part of animal studies was performed under the guidelines of the Institutional Animal Care and Use Committee (12 March 2015). The C57BL/6J mice (male) aged 4 weeks (total amount = 63) were obtained from the National Laboratory Animal Breeding Center. All rodents were haphazardly partitioned to control (CON) group (control diet) (Diet 12450B, Research Diets, Inc.; low-fat diet) (*n* = 9) and high-fat diet (HFD) (Diet 12451, Research Diets, Inc.) group [10, 33, 34]. The low-fat diet was composed of protein 20%, carbohydrate 70%, and fat 10%, whereas high-fat diet was composed of protein 20%, carbohydrate 35%, and fat 45% (of total energy, % kcal). The CON mice were on the control diet, and the HFD mice were on 45% HFD for 12 weeks [33]. The control diet or HFD is comprised of 10% fat or 45% fat, respectively. After HFD-induction for 8 weeks, the HFD-fed group (total amounts: 54 mice) was again divided into 6 groups (*n* = 9, per group) as follows: treatment with AnK (including AnK1: 10, AnK2: 20, and AnK3: 40 mg/kg/day bw), or fenofibrate (Feno: 0.25 g/kg/day bw, Sigma Chemical Co.), or metformin (0.3 g/kg/day bw), or vehicle with oral gavage one time every day for 28 days, and the CON and high-fat control (HF) groups were given only vehicle [10, 33]. After administration of AnK, Feno, or Metf for 4 weeks, the mice (12 h fasting) were sacrificed and peripheral tissues were weighed. Parts of tissues were immediately stored at −80°C for targeted genes analysis. Blood glucose analysis and biochemical parameters (including TG, TC, and FFA), adipocytokine (including insulin, adiponectin, and leptin) levels, and metabolic parameters including body weight, weight gain, and food intake were performed as previous procedures [10, 11, 33].

### 2.6. Assessment of Blood Glucose and Biochemical Parameters

Blood sample was obtained from the retro-orbital sinus of 12 h fasting mice. Blood glucose level (by the glucose oxidase method); plasma TG, TC, and free fatty acids level (using commercial assay kits); and insulin, leptin, and adiponectin level (by enzyme-linked immunosorbent assay (ELISA) kits) were measured as previous reports [11, 33, 35, 36].

### 2.7. Histopathology Examination

Parts of visceral adipose and liver specimen were measured and pictures were taken as previous reports [11, 33, 36].

### 2.8. Analysis of Liver Lipids

This procedure was performed as in previous reports [37].

### 2.9. Relative Quantization of mRNA Indicating Gene Levels and Western Blotting

These procedures of relative quantization of mRNA (the primers are described in Table 1) and immunoblots in the measurement of skeletal muscular GLUT4, phospho-AMPK (Thr^172^)/total-AMPK (Thr^172^), or phospho-Akt (Ser473)/total-Akt (Ser473) proteins from the muscle and liver of mice were performed as previous procedures elsewhere [10, 11, 33, 35, 36]. PPAR*α* and FAS proteins were performed from the liver tissue and PPAR*γ* and FAS proteins from the adipose tissue of mice. Skeletal muscle from mice was subjected to GLUT4 expression level analysis. Total membrane fraction was measured; and the expression levels of GLUT4, phospho-AMPK, and total-AMPK were determined by Western blotting as in described reports [10, 11, 33, 35, 36].

### 2.10. Statistics

Results present means and standard error. Comparisons among groups were using ANOVA and coupled with Dunnett's tests. *P* values less than 0.05 were regarded as statistically significant differences.

## 3. Results

### 3.1. Membrane GLUT4 and Akt Phosphorylation Expression* In Vitro*

**Figure 2:**
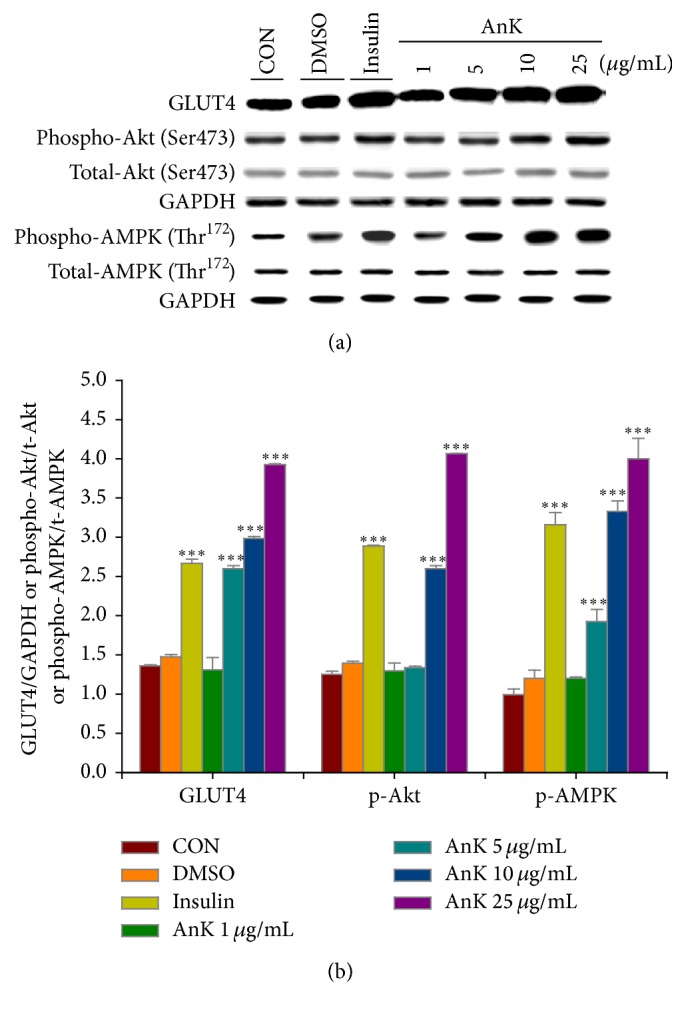
Effect of antcin K (AnK) on GLUT4, phospho-Akt/total-Akt, and phospho-AMPK/total-AMPK* in vitro*. C2C12 myoblasts cells were treated with AnK compounds as described in the experimental procedures and equal amounts of lysates were resolved by SDS PAGE and blotted for GLUT4, Akt, phospho-Akt (Ser473), AMPK, and phospho-AMPK (Thr^172^). (a) Representative blots for AnK in C2C12 myoblasts cells; (b) quantification of the GLUT4 protein contents and the ratio of phospho-Akt to total-Akt and phospho-AMPK to total-AMPK. All values are means ± SE. ^*∗∗∗*^ *P* < 0.001 compared with the control group.

The membrane GLUT4 expressions were higher in the insulin- and AnK-treated (5, 10, and 25 *μ*g/mL) groups than in the CON group. The phospho-Akt (Ser473)/total-Akt expressions were higher in the insulin- and AnK-treated (10 and 25 *μ*g/mL) groups than in CON group (Figures 2(a) and 2(b)).

### 3.2. Metabolic Parameters

At the beginning, the average body weights of all mice were 20.05 ± 0.13 g. At the end, body weight and body weight gain were markedly enhanced in HFD-induced mice (Table 2). AnK2-, AnK3-, or Feno-treated mice had decreased body weight, while AnK1-, AnK2-, AnK3-, Feno-, or Metf- treated groups had decreased body weight gain. The HF mice consume less food intake than CON mice (Table 2). No difference was found in food intake between AnK-, Feno-, or Metf-treated groups and HF group. Feeding a HFD displayed increases in absolute epididymal, mesenteric, retroperitoneal white adipose tissue (WAT) and visceral fat weights (Table 2). The AnK1-, AnK2-, AnK3-, Feno-, or Metf-treated groups reduced epididymal, retroperitoneal WAT, mesenteric WAT, and visceral fat weights. Feno-treated mice showed a decrease in brown adipose tissue (BAT) weights, but increased weights of the liver (Table 2).

### 3.3. Fasting Blood Glucose Levels, Biochemical Parameters, Adipocytokine Levels, and Liver Lipids

**Figure 3:**
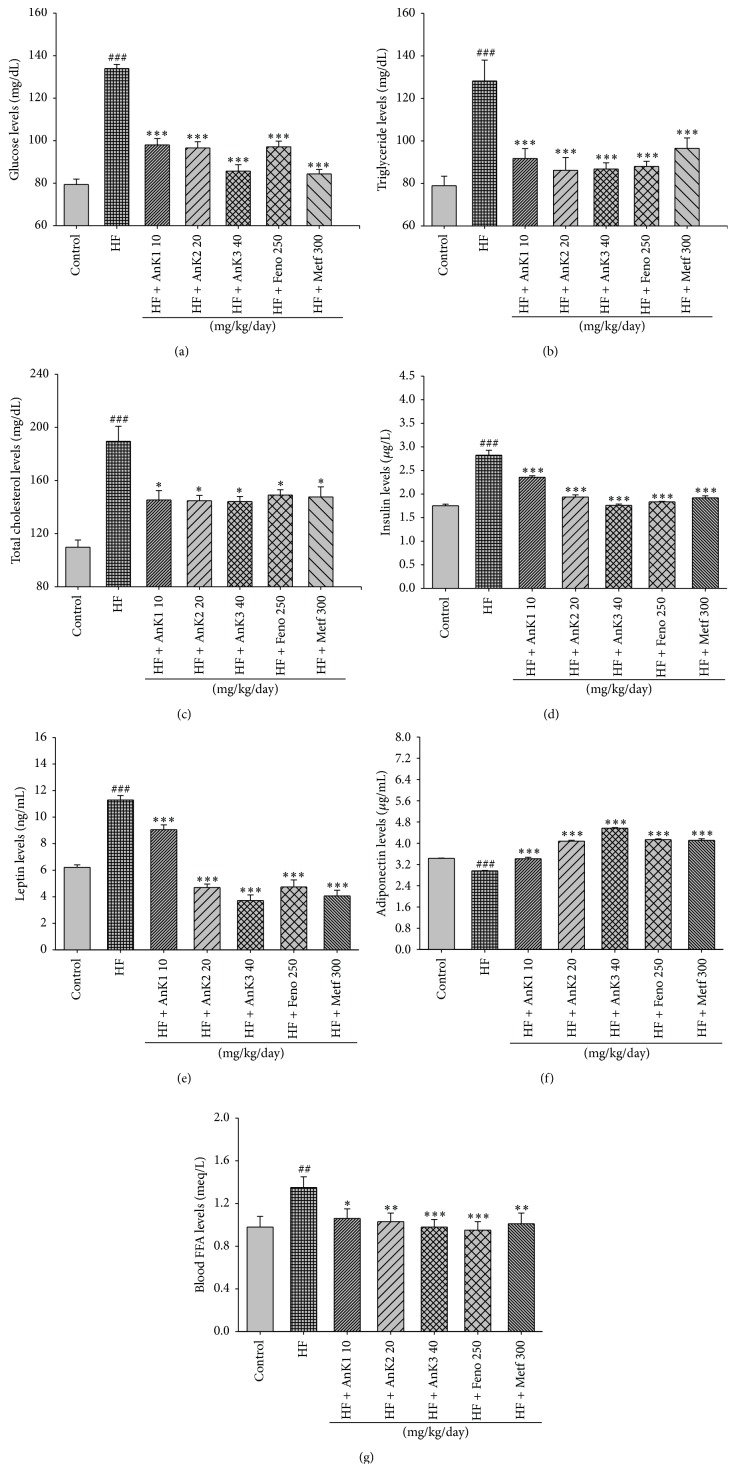
Effects of antcin K (AnK) on (a) blood glucose levels, (b) blood triglycerides levels, (c) blood total cholesterol levels, (d) insulin levels, (e) leptin levels, (f) adiponectin levels, and (g) blood FFA levels at week 12. Mice were fed with 45% high-fat diet (HF) or low-fat diet (CON) for 12 weeks. After 8 weeks of induction, the HF mice were treated with vehicle, or antcin K, or fenofibrate (Feno), or metformin (Metf) accompanied with HF diet for 4 weeks. All values are means ± SE (*n* = 9). ^##^ *P* < 0.01 and ^###^ *P* < 0.001 compared with the control (CON) group; ^*∗*^ *P* < 0.05, ^*∗∗*^ *P* < 0.01, and ^*∗∗∗*^ *P* < 0.001 compared with the high-fat diet plus vehicle (distilled water) (HF) group by ANOVA. AnK (AnK1, AnK2, or AnK3, 10, 20, or 40 mg/kg body wt); fenofibrate (Feno, 250 mg/kg body wt); metformin (Metf, 300 mg/kg body wt). FFA, plasm free fatty acid; visceral fat represented epididymal WAT plus retroperitoneal WAT.

It is evident that hyperglycemia has been observed after 12 weeks of HFD treatment (*P* < 0.001). Treatment with AnK1, AnK2, AnK3, Feno, and Metf markedly lowered glucose levels in blood (Figure 3(a)). HFD increased the levels of circulating TG, total cholesterol (TC), and free fatty acid (Figures 3(b) and 3(c) and Table 2). The AnK1-, AnK2-, AnK3-, Feno-, or Metf-treated mice had decreased TG, TC, and FFA levels. Plasma insulin and leptin concentrations were higher, but adiponectin levels were lower in the HF group than in the CON group. The AnK1-, AnK2-, AnK3-, Feno-, and Metf-treated mice had effectively reduced plasma leptin, insulin, and FFA concentrations but markedly enhanced adiponectin levels (Figures 3(d), 3(e), 3(f), and 3(g)). HFD enhanced the levels of liver total lipids and triacylglycerol, and AnK1-, AnK2-, AnK3-, Feno-, or Metf-treated mice had decreased hepatic total lipid and triacylglycerol levels (Table 2).

### 3.4. Histopathology Examination

**Figure 4:**
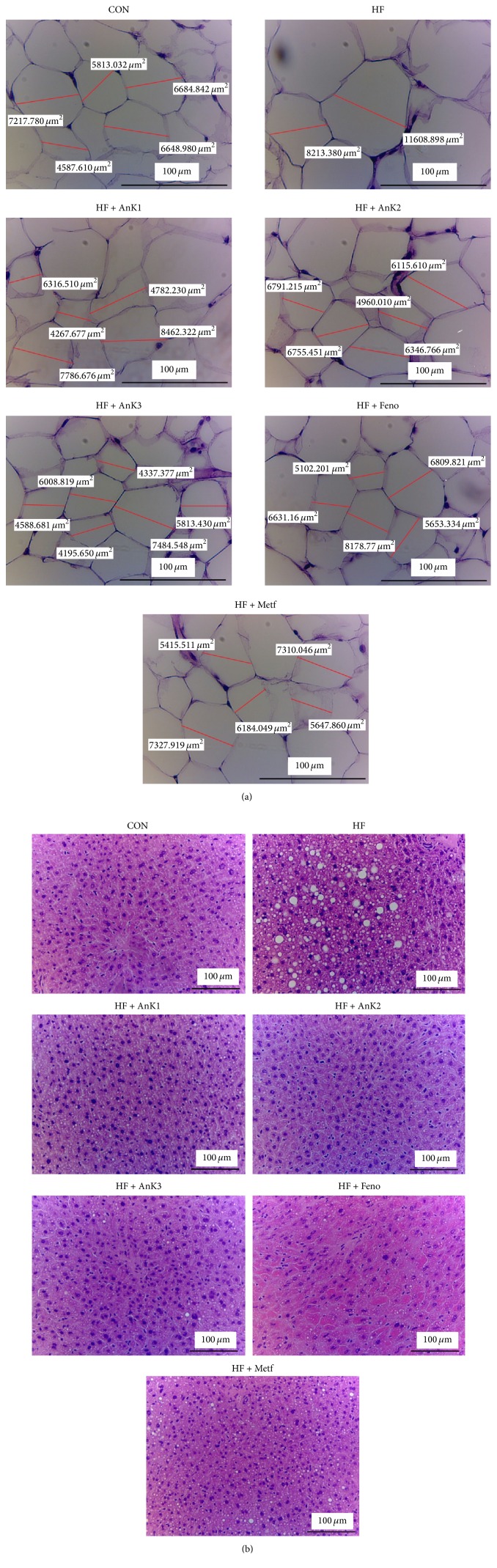
Histology of (a) epididymal white adipose tissue and (b) liver tissue of mice in the control (CON), high-fat diet plus vehicle (distilled water) (HF), HF + AnK1, HF + AnK2, HF + AnK3, HF + fenofibrate (Feno), or HF + metformin (Metf) groups by hematoxylin and eosin-staining. Magnification: 10 (ocular) × 20 (object lens). Antcin K (AnK1, AnK2, or AnK3, 10, 20, or 40 mg/kg body weight, resp.); Feno, fenofibrate (250 mg/kg body weight). Metf, metformin (300 mg/kg body weight).

HFD caused adipocytes hypertrophy (the following data were calculated average areas: the CON mice, 6044.4 ± 359.1 *μ*m^2^; the HF group, 10142.9 ± 428.1) and following treatment with AnK1 (6548.6 ± 214.7 *μ*m^2^), AnK2 (6483.8 ± 319.8 *μ*m^2^), AnK3 (5670.8 ± 281.6 *μ*m^2^), Feno (6304.2 ± 316.9 *μ*m^2^), or Metf (5873.7 ± 345.1 *μ*m^2^) displayed less hypertrophy (Figure 4(a)). On the basis of a previous study [38], the designation of histological hepatocellular ballooning findings is comprised of grade 0, none; grade 1, few cells; grade 2, many cells. As shown in Figure 4(b), HFD induced the ballooning of hepatocyte (mean score, 1.9 ± 0.1) as compared with the CON group (0) in liver tissue. Administration of AnK1 (0.7 ± 0.2), AnK2 (0.5 ± 0.2), AnK3 (0.4 ± 0.2), Feno (0.5 ± 0.1), or Metf (0.7 ± 0.2) decreased the ballooning as compared with the HF group.

### 3.5. Hepatic Targeted Gene mRNA Levels

**Figure 5:**
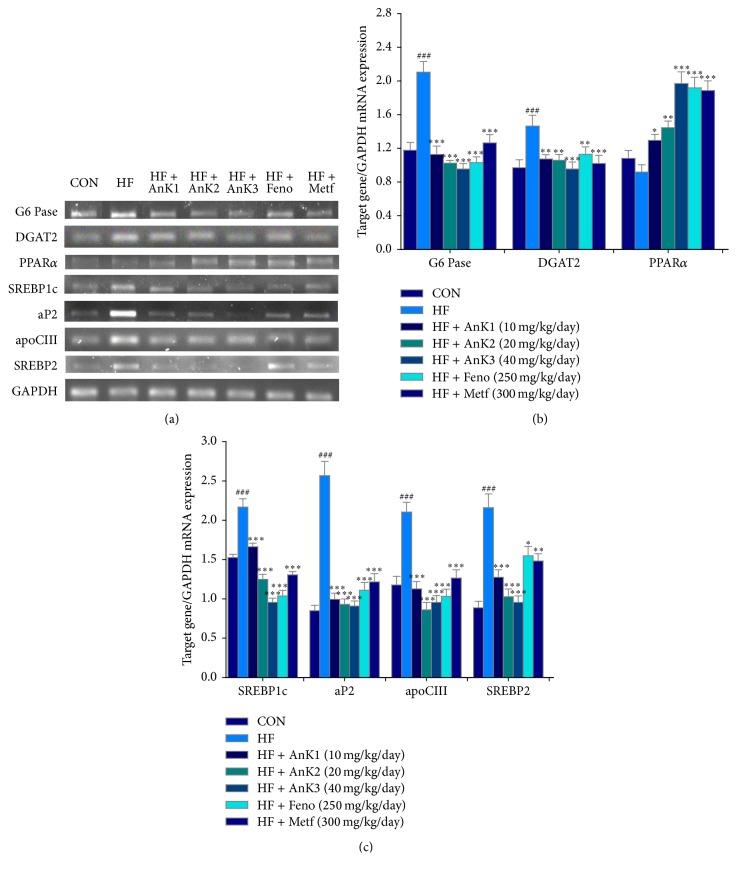
Semiquantitative RT-PCR analysis on G6 Pase, DGAT2, PPAR*α*, SREBP1c, aP2, apoCIII, and SREBP2 mRNA levels in liver tissue of the mice by oral gavage antcin K (AnK1, AnK2, or AnK3, 10, 20, or 40 mg/kg body weight, resp.); Feno, fenofibrate (250 mg/kg body weight); Metf, metformin (300 mg/kg body weight): (a) representative image; (b, c) quantification of the ratio of target gene to GAPDH mRNA expression. Total RNA (1 *μ*g) isolated from tissue was reverse-transcribed by MMLV-RT; 10 *μ*L of RT products was used as templates for PCR. The expression levels of G6 Pase, DGAT2, PPAR*α*, SREBP1c, aP2, apoCIII, and SREBP2 mRNA were measured and quantified by image analysis. Values were normalized to GAPDH mRNA expression. All values are means ± SE (*n* = 9). ^###^ *P* < 0.001 compared with the control (CON) group; ^*∗*^ *P* < 0.05, ^*∗∗*^ *P* < 0.01, and ^*∗∗∗*^ *P* < 0.001 compared with the high-fat-diet plus vehicle (distilled water) (HF) group.

HFD elicits increases in G6 Pase, acyl-coenzyme A: diacylglycerol acyltransferase 2 (DGAT 2), SREBP1c, aP2, apolipoprotein CIII (apo CIII), and SREBP2 mRNA levels. The AnK1-, AnK2-, AnK3-, Feno-, or Metf-treated mice had decreased mRNA levels of G6 Pase, DGAT2, SREBP1c, aP2, apo CIII, and SREBP2 mRNA levels but increased PPAR*α* mRNA levels (Figure 5).

### 3.6. Targeted Protein Expression Levels in Different Tissues

**Figure 6:**
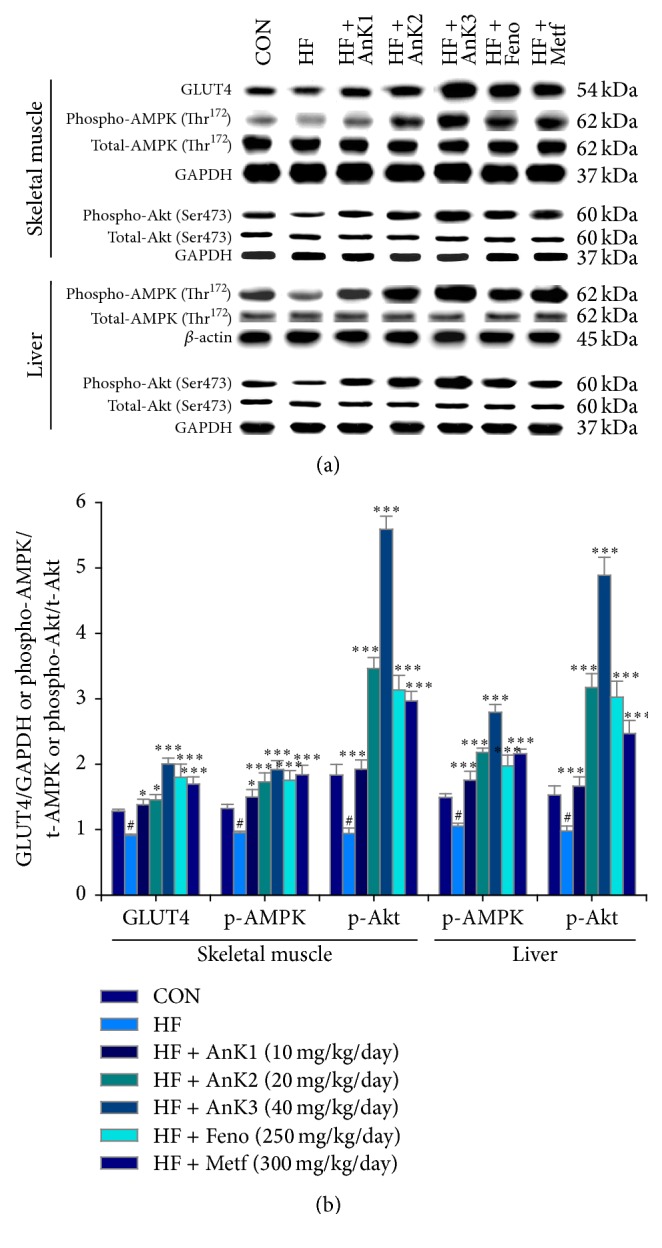
GLUT4 protein contents in skeletal muscle or phospho-Akt/t-Akt or phospho-AMPK (Thr^172^)/t-AMPK in liver and skeletal muscle of the mice by oral gavage antcin K (AnK): (a) representative image; (b) quantification of the GLUT4 expression levels, the ratio of phospho-AMPK to total-AMPK, or phospho-Akt/t-Akt expression levels (mean ± SE, *n* = 9). Protein was separated by 12% SDS PAGE detected by Western blot. ^#^ *P* < 0.05 compared with the control (CON) group; ^*∗*^ *P* < 0.05 and ^*∗∗∗*^ *P* < 0.001 compared with the high-fat-diet plus vehicle (distilled water) (HF) group. Antcin K (AnK1, AnK2, or AnK3, 10, 20, or 40 mg/kg body weight, resp.); Feno, fenofibrate (250 mg/kg body weight); Metf, metformin (300 mg/kg body weight).

**Figure 7:**
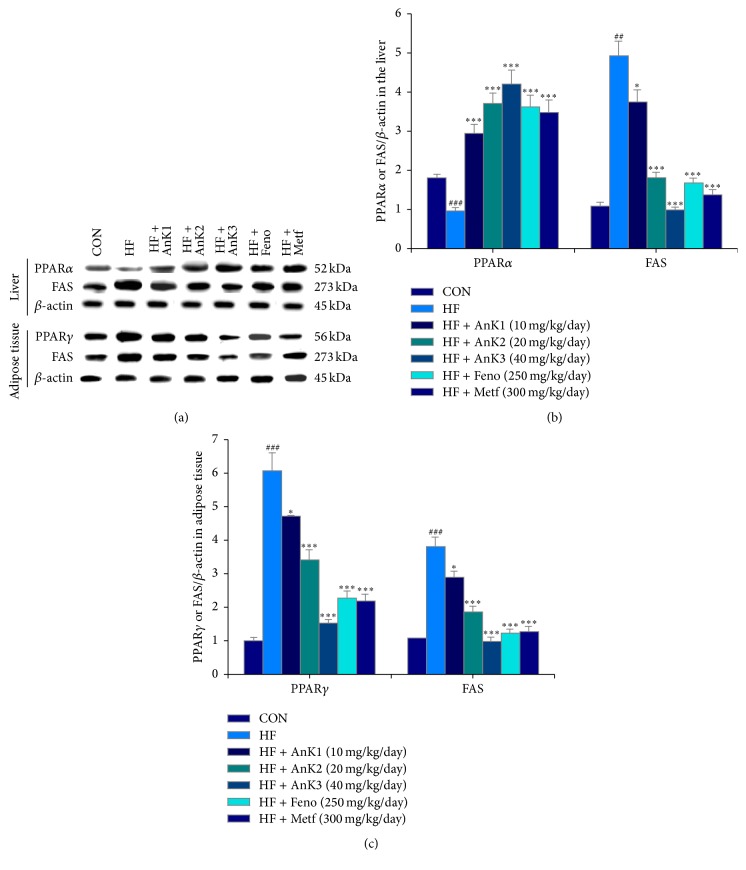
Expression levels of PPAR*α* and FAS in the liver tissue and PPAR*γ* and FAS in adipose tissue of mice by oral gavage antcin K: (a) representative image; (b, c) quantification of the expression levels of PPAR*α* and FAS in the liver tissue and PPAR*γ* and FAS in adipose tissue. Protein was separated by 12% SDS PAGE detected by Western blot. ^##^ *P* < 0.01 and ^###^ *P* < 0.001 compared with the control (CON) group; ^*∗*^ *P* < 0.05 and ^*∗∗∗*^ *P* < 0.001 compared with the high-fat-diet plus vehicle (distilled water) (HF) group. Antcin K (AnK1, AnK2, or AnK3, 10, 20, or 40 mg/kg body weight, resp.); Feno, fenofibrate (250 mg/kg body weight); Metf, metformin (300 mg/kg body weight).

HFD induced decreases in protein expression levels of skeletal muscular membrane GLUT4 (*P* < 0.001). AnK1-, AnK2-, AnK3-, Metf-, or Feno-treated groups enhanced membrane GLUT4 expressions. HFD-induced mice had decreased expression levels of phospho-AMPK/total-AMPK or phospho-Akt/total-Akt in both muscle and the liver, which were markedly enhanced in the AnK1-, AnK2-, AnK3-, Metf-, or Feno-treated mice (Figure 6). HFD-fed mice had decreased liver PPAR*α* expressions, but increased in FAS levels. Treatment with AnK1, AnK2, AnK3, Feno, or Metf increased PPAR*α* but decreased FAS expression levels in the liver (Figure 6). The adipose PPAR*γ* and FAS expressions were increased in the HF group. Treatment with AnK1, AnK2, AnK3, Feno, or Metf decreased PPAR*γ* and FAS expression levels in adipose tissue (Figure 7).

## 4. Discussion

Skeletal muscle and adipose tissue play unique roles in the regulation of insulin-dependent glucose homeostasis [39]. Skeletal muscle is proposed to be the primary site of whole-body insulin-mediated glucose uptake [15, 40, 41]. Adipose tissue accounts for a small fraction of glucose disposal after a meal, with the majority of glucose uptake by muscles [41, 42]. Therefore, this study was firstly designed to screen GLUT4 protein expression in* in vitro* myotubes. And we knew that if* in vitro* study of the compound displays effectiveness, it cannot be assumed to have the same effect* in vivo*, since it entered physical body and underwent biotransformation including absorption, distribution, metabolism, and excretion. Thus, this study was focused on performance of targeted gene protein expressions in different tissues of AnK-treated HFD-fed mice. This study firstly observed that AnK treatment at 5, 10, and 25 *μ*g/mL* in vitro* significantly increased membrane expression levels of GLUT4 in C2C12 myoblast cells. We further undertake to assess whether AnK exhibit antidiabetic and antihyperlipidemic activity employing the HFD animal model since insulin resistance plays the majority of all diabetes cases and to compare with the antidiabetic drug, metformin, and the hypolipidemic drug, fenofibrate, which has also been shown to display good glycemic control [43]. Here we observed that HFD-induction was in line with the previous observation displaying increases in blood glucose, triglyceride, total cholesterol, insulin, and leptin levels [26]. After the treatment, AnK exhibited both antidiabetic and antihyperlipidemic effects in HFD-fed mice. AnK-treated mice show the glucose-lowering effect by 26.8%–36.0%. Of interest, the glucose-lowering effect of AnK at 40 mg/kg (with less than one-seventh of Meft dosage) was comparable to that of metformin. Our results demonstrated that AnK display good antidiabetic activities; moreover, AnK treatment decreased blood insulin levels and finally improved HFD- induced insulin resistance. These favorable antidiabetic effects of AnK were owing to enhancement of insulin sensitivity in peripheral tissues, particularly increased membrane GLUT4 expressions in skeletal muscle and enhanced activation of AMPK in muscle and the liver.

In addition, all of the AnK-treated groups decreased circulating triglyceride concentrations by 28.5%–32.8% comparable to that of Feno, which is a PPAR*α* agonist with triglyceride-lowering effect [43]. The overall effects in HFD-fed mice imply that AnK had therapeutic potential for the management of type 2 diabetes accompanied with hyperlipidemia.

The first aim of this study was undertaken to assess muscular membrane GLUT4 expressions following treating HFD-fed mice with AnK. Skeletal muscle plays the major site of whole-body insulin-mediated glucose uptake [15]. The membrane GLUT4 expressions measured the translocation of insulin responsive glucose transporter GLUT4 to the plasma membrane [19]. In this study, treatment with AnK, Feno, or Metf significantly increased membrane expression levels GLUT4 by 1.52–2.20-, 1.98-, or 1.86- fold as compared with the HF group, respectively, implying that the increased membrane GLUT4 contents are enhanced to cause glucose uptake, resulting in a decrease in blood glucose levels.

Evidence suggests that the C2C12 myotube is a useful model for analyzing GLUT4 translocation in skeletal muscle [44]. Akt (PKB) stimulates glucose uptake by modulating glucose transporter 4 (GLUT4) [45]. The promoted glucose uptake into skeletal muscle included two pathways: insulin-dependent mechanisms lead to activation of Akt and contraction-mediated stimulation of AMPK [12, 17]. In this* in vitro* experiment, our results showed that AnK (between 1 and 25 *μ*g/mL) was not toxic to C2C12 myotubes by employing the MTT (3-[4,5-dimethylthiazol-2-yl]-2,5-diphenyltetrazolium bromide) assay (data not shown), and AnK significantly enhanced membrane GLUT4 proteins and phospho-AMPK/total-AMPK expressions at 5, 10, and 25 *μ*g/mL and enhanced phospho-Akt/total-Akt expressions at 10 and 25 *μ*g/mL, and we assume that AnK in myotube cells at 10 and 25 *μ*g/mL could stimulate glucose transport activity partly by insulin pathway and partly by AMPK activation.

The second aim of this study was to evaluate the phospho-AMPK protein expression in AnK-treated HFD-fed mice, since AMPK plays the core role of glucose and lipid metabolism. These data showed that AnK treatment increased the expressions of phospho-AMPK/total-AMPK in the muscle and liver. Metformin may enhance skeletal muscular AMPK activity [17, 46]. Chronic activation of AMPK may induce GLUT4 deployment to the plasma membrane, leading to insulin-independent glucose uptake [17, 46, 47]. In skeletal muscle, AnK was found to increase AMPK phosphorylation comparable to that of metformin, suggesting that AnK activates AMPK or Akt phosphorylation to increase GLUT4 translocation in muscles, which leads to a decrease in systemic insulin resistance.

G6 Pase plays a key role in gluconeogenesis [48]. The hepatic expression of mRNA level of G6 Pase is reduced in AnK-treated mice. Collectively, our results imply that AnK display glucose-lowering effects via enhanced muscular GLUT4 proteins to increase glucose uptake and decreased hepatic G6 Pase mRNA levels to suppress hepatic glucose production.

The third aim of this study was to clarify the hypolipidemic effects and mechanisms of AnK. Evidences have shown that PPAR*α* are abundantly expressed in the liver tissue and promoted fatty acids oxidation [49]. PPAR*α* agonists have been proposed as a breakthrough in the management of dyslipidemia to reduce blood triglyceride levels [43, 49]. In this study, AnK displayed antihypertriglyceridemic effects. PPAR*α* ligands could reduce the expression of the apo CIII gene [50], thus resulting in hypotriglyceridemic effect. DGAT2 play a role in the final step of triglyceride synthesis [51]. SREBP-1c, a key lipogenic transcription factor, stimulates lipogenic enzyme expression and contributes to fatty acids synthesis and TG accumulation [52]. Mice with aP2 deficiency are protected from the development of dyslipidemia, hyperglycemia, insulin resistance, and fatty liver disease in both genetic and dietary obesity [53]. Ablation of aP2 and mall show enhanced liver accumulation of longer-chain fatty acids, thus resulting in decreased SREBP1c expressions and its several downstream lipogenic enzymes [53]. We found that hepatic mRNA levels of aP2 and lipogenic SREBP1c are suppressed by AnK, thus also contributing to protecting from HFD-induced insulin resistance and hepatic steatosis. FAS is a critical focus in fatty acid synthesis [54]. SREBP2 play a core role in the regulation of cholesterol synthesis [55]. AnK lowered plasma TC concentrations coincident with reduced SREBP2 mRNA levels, implying AnK exerting TC-lowering effect may be primarily due to a decrease of cholesterol synthesis. Taken together, AnK-treated mice had increased hepatic expression of PPAR*α* protein to enhance fatty acids oxidation but decreased FAS protein to inhibit fatty acids synthesis coincident with suppressed SREBP1c, aP2, DGAT2, and apo CIII mRNAs, thus contributing to the hepatic triglyceride output and leading to decreased plasma triglycerides, hepatic steatosis, and total cholesterol levels.

In adipose tissue, PPAR*γ* stimulated adipogenesis and lipogenesis [56]. PPAR*γ* is abundantly expressed in adipocytes and its expression is markedly induced during adipocyte differentiation [57]. Here we report that treatment with AnK, Feno, or Metf decreased adipose expression of PPAR*γ* and FAS protein; as a result, adipogenesis and fatty acids synthesis and lipid accumulation are reduced in adipose tissue. Moreover, blood TG is fluctuating between the liver and adipose tissue. Lipid could usually be stored in the adipose tissue and the liver is the major organ of lipid metabolism, presuming AnK could remove fat from adipose tissue to peripheral tissues not only by increasing lipid catabolism including inhibition of fatty acid synthesis (FAS) and enhancement of fatty acid oxidation (PPAR*α*) in the liver, but also by inhibition of adipocyte adipogenesis (PPAR*γ*) and FAS in adipose tissue, thus leading to reduced TG levels in the liver, blood, and adipose tissue. Therefore, in histology analysis, AnK treatment resulted in a decrease in lipid accumulation in adipose tissue and liver and finally reflected hepatic lipid drops almost invisible and a reduction in adipocyte size.

Adiponectin level was found to decrease in HFD-fed mice in this study. This observation is in line with the others demonstrating that adiponectin levels are reduced in adults or rodents with obesity and type 2 diabetes [58]. High levels of adiponectin can predict enhanced insulin sensitivity of both glucose and lipid metabolism [59]. Following AnK administration, the mice display significantly increased blood levels of adiponectin, establishing that AnK could provide a unique therapeutic advantage associated with the regulation to improve insulin sensitivity. Moreover, studies have showed that there is an inverse relationship between plasma leptin or mRNA expression of leptin and insulin sensitivity [60]. In this study, leptin level is enhanced in HFD-fed mice, in accordance with a previous report [61]. Treatment with AnK markedly reduced the increase of leptin level. Thus, AnK prevented HFD-induced abnormalities in leptin levels and improved insulin resistance. Treatment with globular domain of adiponectin increased glucose uptake and AMPK activation [62]. Adiponectin is proposed to activate AMPK in the liver, enhance glucose utilization and fatty acid oxidation, and inhibit glucose production in the liver [63]. Administration of AnK significantly elevated phosphorylation of AMPK. On the basis of the previous reports [62, 64], the AMPK phosphorylation by AnK may be associated with adiponectin and/or leptin secretion. Thus, there is possibility that AnK directly cause AMPK phosphorylation or act by adiponectin-mediated activation of AMPK and PPAR*α* leads to a reduction in hepatic gluconeogenesis and increased muscle glucose uptake, resulting in reduced glucose levels* in vivo* and increased fatty acid oxidation in both tissues.

**Figure 8:**
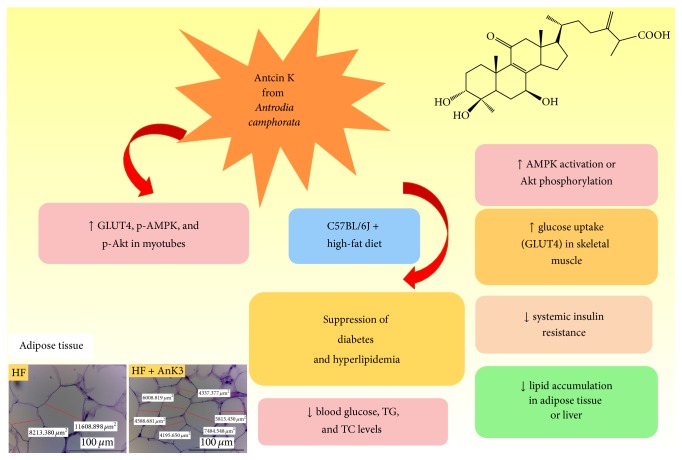
A proposed mechanism for AnK to improve diabetes and hyperlipidemia.

In conclusion, AnK-treated mice had not only lowered blood glucose and insulin, but also decreased triglyceride, total cholesterol levels, and finally ameliorated insulin resistance (Figure 8). Of interest, AnK at 40 mg/kg/day dosage displayed both antihyperglycemic effect comparable to Metf (300 mg/kg/day) and antihypertriglyceridemic effect comparable to Feno (250 mg/kg/day). The antidiabetic effect of AnK is due to significant increases in membrane GLUT4 expression levels in skeletal muscle to stimulate glucose uptake coincident with decreases in G6 Pase mRNA levels to inhibit hepatic glucose production, thus contributing to glucose-lowering efficacy. In both skeletal muscle and liver tissue, AnK-treated mice had increased AMPK activation. AnK treatment exhibited inhibition of hepatic lipogenic FAS expression but enhancement of fatty acid oxidation PPAR*α* expression coincident with reduced SREBP1c mRNA levels in the liver, thus resulting in decreased plasma triglycerides and total cholesterol levels. AnK activates AMPK or Akt phosphorylation to increase GLUT4 translocation in muscles, which leads to a decrease in systemic insulin resistance and to fat accumulation in adipose tissue and liver. Additionally, the ameliorated insulin resistance also improved the liver insulin sensitivity (Akt activation). Our findings manifest that AnK has a favorable therapeutic potential for the management of type 2 diabetes associated with hyperlipidemia.

## Supplementary Material

Figure 1. The HPLC analysis of Antcin K (AnK), and it was observed that in addition to AnK, there is no any other compound to exist.
Figure 2. The NMR analysis of Antcin K (AnK) was eluted with a pyridine-d5 solvent.
Figure 3. The NMR analysis of Antcin K (AnK) was eluted with a MeOH solvent.

## Figures and Tables

**Table 1:** Primers used in this study.

Gene	Accession number	Forward primer and reverse primer	PCR product (bp)	Annealing temperature (°C)
Liver				
G6 Pase	NM_008061.3	F: GAACAACTAAAGCCTCTGAAAC R: TTGCTCGATACATAAAACACTC	350	50
SREBP1c	NM_011480	F: GGCTGTTGTCTACCATAAGC R: AGGAAGAAACGTGTCAAGAA	219	48
DGAT2	NM_026384.3	F: AGTGGCAATGCTATCATCATCGT R: AAGGAATAAGTGGGAACCAGATCA	149	50
apo C-III	NM_023114.3	F: CAGTTTTATCCCTAGAAGCA R: TCTCACGACTCAATAGCTG	349	47
SREBP2	AF289715.2	F: ATATCATTGAAAAGCGCTAC R: ATTTTCAAGTCCACATCACT	256	48
PPAR*α*	NM_011144	F: ACCTCTGTTCATGTCAGACC R: ATAACCACAGACCAACCAAG	352	49
aP2	NM_024406	F: TCACCTGGAAGACAGCTCCT R: TGCCTGCCACTTTCCTTGT	142	52
GAPDH	NM_008084.3	F: TGTGTCCGTCGTGGATCTGA R: CCTGCTTCACCACCTTCTTGA	99	55

**Table 2:** Effects of antcin K (AnK) on tissue weight, food intake, and liver lipid.

Dose (mg/kg/day)	CON	HF	HF + AnK1	HF + AnK2	HF + AnK3	HF + Feno	HF + Metf
			10	20	40	250	300
Absolute tissue weight (g)							
EWAT	0.531 ± 0.052	1.264 ± 0.147^###^	0.867 ± 0.065^*∗∗*^	0.841 ± 0.062^*∗∗*^	0.809 ± 0.058^*∗∗∗*^	0.603 ± 0.041^*∗∗∗*^	0.813 ± 0.064^*∗∗∗*^
MWAT	0.278 ± 0.031	0.439 ± 0.025^###^	0.349 ± 0.020^*∗*^	0.340 ± 0.013^*∗*^	0.332 ± 0.025^*∗*^	0.247 ± 0.025^*∗∗∗*^	0.270 ± 0.018^*∗∗∗*^
RWAT	0.166 ± 0.021	0.483 ± 0.064^###^	0.323 ± 0.039^*∗*^	0.339 ± 0.031^*∗*^	0.306 ± 0.040^*∗*^	0.181 ± 0.020^*∗∗∗*^	0.298 ± 0.027^*∗∗*^
Visceral fat	0.697 ± 0.056	1.747 ± 0.208^###^	1.190 ± 0.093^*∗∗*^	1.180 ± 0.106^*∗∗*^	1.154 ± 0.096^*∗∗∗*^	0.784 ± 0.052^*∗∗∗*^	1.111 ± 0.077^*∗∗∗*^
Skeletal muscle	0.308 ± 0.014	0.412 ± 0.045	0.395 ± 0.036	0.364 ± 0.022	0.364 ± 0.028	0.428 ± 0.026	0.380 ± 0.025
BAT	0.158 ± 0.004	0.224 ± 0.022^#^	0.178 ± 0.007	0.172 ± 0.010	0.175 ± 0.008	0.157 ± 0.013^*∗*^	0.220 ± 0.025
Liver (g)	1.003 ± 0.024	0.987 ± 0.029	0.946 ± 0.030	0.888 ± 0.019	0.883 ± 0.018	1.700 ± 0.070^*∗∗∗*^	0.908 ± 0.031
Spleen (g)	0.099 ± 0.006	0.094 ± 0.004	0.090 ± 0.003	0.085 ± 0.003	0.104 ± 0.007	0.084 ± 0.005	0.093 ± 0.006
Final body weight (g)	27.21 ± 0.47	30.43 ± 1.02^#^	28.30 ± 0.61	27.55 ± 0.72^*∗*^	27.48 ± 0.46^*∗*^	27.55 ± 0.84^*∗*^	27.86 ± 0.72
Weight gain (g)	1.61 ± 0.15	3.42 ± 0.24^#^	1.39 ± 0.81^*∗*^	0.70 ± 0.86^*∗∗*^	0.58 ± 0.35^*∗∗*^	0.57 ± 0.55^*∗∗∗*^	0.92 ± 0.08^*∗∗*^
Food intake (g/day/mouse)	2.34 ± 0.04	1.99 ± 0.04^###^	1.95 ± 0.05	1.92 ± 0.07	1.98 ± 0.04	1.99 ± 0.06	1.89 ± 0.04

Liver lipids							
Total lipid (mg/g)	53.7 ± 2.7	95.9 ± 6.4^###^	73.1 ± 4.7^*∗∗*^	66.0 ± 4.8^*∗∗*^	64.5 ± 5.2^*∗∗*^	64.9 ± 5.1^*∗∗*^	65.3 ± 4.9^*∗∗*^
Triacylglycerol (*μ*mol/g)	40.6 ± 3.9	79.3 ± 6.3^###^	56.3 ± 4.2^*∗∗*^	45.7 ± 3.9^*∗∗∗*^	45.2 ± 4.6^*∗∗∗*^	47.3 ± 4.6^*∗∗∗*^	45.4 ± 4.2^*∗∗∗*^

Antcin K (AnK; AnK1, AnK2, and AnK3, 10, 20, and 40 mg/kg body wt); fenofibrate (Feno, 250 mg/kg body wt); metformin (Metf, 300 mg/kg body wt); BAT, brown adipose tissue; skeletal muscle included quadriceps muscle, which contains four parts, rectus femoris, vastus intermedius, vastus lateralis, and vastus medialis. All values are means ± SE (*n* = 9). ^#^ *P* < 0.05 and ^###^ *P* < 0.001 compared with the control (CON) group; ^*∗*^ *P* < 0.05, ^*∗∗*^ *P* < 0.01, and ^*∗∗∗*^ *P* < 0.001 compared with the high-fat plus vehicle (distilled water) (HF) group. Epididymal white adipose tissue (epididymal WAT; EWAT), retroperitoneal WAT (RWAT), and mesenteric WAT (MWAT). Visceral fat represented epididymal WAT plus retroperitoneal WAT.

## Abbreviations

AMPK: AMP-activated protein kinase
aP2: Adipocyte fatty acid binding protein 2
BAT: Brown adipose tissue
CON: Control
DGAT2: Acyl-coenzyme A: diacylglycerol acyltransferase 2
EWAT: Epididymal white adipose tissue
FAS: Fatty acid synthase
Feno: Fenofibrate
FFA: Free fatty acid
GAPDH: Glyceraldehyde-3-phosphate dehydrogenase
G6 Pase: Glucose-6-phosphatase
GLUT4: Glucose transporter 4
HF: High-fat control
HFD: High-fat diet
Metf: Metformin
MWAT: Mesenteric white adipose tissue
PPAR: Peroxisome proliferator-activated receptor
RT-PCR: Reverse transcription-polymerase chain reaction
RWAT: Retroperitoneal white adipose tissue
SREBP: Sterol regulatory element binding protein
TC: Total cholesterol
TG: Triglyceride
WAT: White adipose tissue.
