# Supplementary material for: Antcin K, a Triterpenoid Compound from Antrodia camphorata, Displays Antidiabetic and Antihyperlipidemic Effects via Glucose Transporter 4 and AMP-Activated Protein Kinase Phosphorylation in Muscles
Source: Evid Based Complement Alternat Med. 2016 May 8;2016:4867092. doi: 10.1155/2016/4867092 (PMC4875994; doi:10.1155/2016/4867092)
Supplement: Supplementary file 1 — Figure 1. The HPLC analysis of Antcin K (AnK), and it was observed that in addition to AnK, there is no any other compound to exist. Figure 2. The NMR analysis of Antcin K (AnK) was eluted with a pyridine-d5 solvent. Figure 3. The NMR analysis of Antcin K (AnK) was eluted with a MeOH solvent. [file 4867092.f1.doc]

**Sample name :Antcin K**

**Instrument:**

**HPLC:** SHIMADZU LC 20-A

**HPLC Column:** TOSOH **TSKgel ODS-80Ts**

100％MeOH

Analysis of results:

Antcin K

solvent peak

(Nuclear Magnetic Resonance Spectroscopy ,NMR): Bruker DMX-500 MHz

NMR Spectrometer Solvent: Pyridine-d5

NMR Spectrometer Solvent: MeOD-d4 (Deuterated methanol)
